# The burden and its determinants of mental health distress among adolescents dwelling in Africa: a systematic review and meta-analysis

**DOI:** 10.1186/s13034-024-00782-4

**Published:** 2024-07-18

**Authors:** Techilo Tinsae, Shegaye Shumet, Girmaw Medfu Takelle, Gidey Rtbey, Mamaru Melkam, Fantahun Andualem, Girum Nakie, Tesfaye Segon, Selam Koye, Setegn Fentahun, Wondale Getinet Alemu, Gebresilassie Tadesse

**Affiliations:** 1 College of Medicine and Health Science, Department of Psychiatry, University of Gondar, Gondar, Ethiopia; 2 Department of Psychiatry, Mettu University, Metu, Ethiopia

**Keywords:** Adolescents, Mental health distress, Mental health distress, Emotional distress, And Africa

## Abstract

**Background:**

Adolescent mental health issues are emerging as a significant public health concern across many low- and middle-income countries, particularly in Africa. This study aims to evaluate the aggregated prevalence and contributing risk factors of mental health distress among adolescents in Africa.

**Methodology:**

A comprehensive search of PubMed, PsycINFO, Web of Science, Google Scholar, and HINARI databases was conducted to identify relevant articles on the prevalence and risk factors associated with mental health distress among African adolescents, published up to December 2023. The quality of the selected studies was assessed using the Newcastle-Ottawa Quality Assessment Scale. Heterogeneity among the studies was evaluated using the I² statistical test. Potential publication bias was assessed through a funnel plot and Egger’s statistical test. This systematic review was registered with PROSPERO under reference number CRD42023494665.

**Results:**

Eighteen studies encompassing data from 37,016 adolescents were included in the analysis. The overall prevalence of mental health distress among adolescents in Africa was found to be 27.34% (95% CI: 23.18–31.50). The occurrence of mental health distress is observed in older adolescents at a prevalence of 29.44% (95% CI: 23.26–35.66) and in younger adolescents at 24.73% (95% CI: 11.96–37.51). Significant risk factors identified included bullying victimization, with an odds ratio (POR) of 1.30 (95% CI: 1.16, 1.46), and experiencing hunger, with an odds ratio (POR) of 2.10 (95% CI: 1.13, 3.91).

**Conclusion:**

The findings indicate a high prevalence of mental health distress among adolescents in Africa, highlighting the widespread impact on this demographic. These results underscore the urgent need for targeted interventions to prevent and address mental health distress among adolescents. Further research on a global scale is essential to develop effective prevention and treatment strategies tailored to this age group.

**Supplementary Information:**

The online version contains supplementary material available at 10.1186/s13034-024-00782-4.

## Introduction

According to the World Health Organization (WHO), adolescence is a critical phase of human development that spans from ages 10 to 19 [1, 2]. This period is marked by rapid physical growth, emotional changes, and significant psychological development [3]. Adolescents navigate the transition from childhood to adulthood, acquiring new social roles and responsibilities [3]. They experience profound biological changes, such as puberty, that influence their emotional and cognitive growth [4]. This stage is also characterized by increased independence and identity formation, as young people begin to explore their personal beliefs, values, and aspirations. Understanding the unique characteristics of adolescents is crucial for supporting their health and well-being during this transformative period [5].

Mental health distress is a condition of emotional suffering characterized by the undifferentiated manifestations of anxiety and depression symptoms, such as restlessness and tenseness, as well as somatic symptoms, such as headaches, insomnia, and low energy [6–9]. According to the stress-distress model, mental health distress arises from encountering a stressful situation that threatens physical or mental well-being, an inability to manage this stress effectively, and the subsequent emotional turmoil caused by ineffective coping mechanisms [10]. “Adolescence is a crucial time for promoting psychological well-being and early mental health intervention to safeguard against the development of mental health issues, as it is characterized by vulnerability to mental health distress [11–13]. " Mental health problems among adolescents are increasingly recognized as a major public health issue in many low- and middle-income countries (LMICs), particularly across Africa [14, 15]. Worldwide, between 10% and 20% of adolescents encounter severe mental health issues [16]. Approximately half (50%) of all instances of mental illness in adults originate during adolescence [17]. Among individuals aged 10 to 19, mental health disorders, including anxiety and depression, contribute to 16% of the global burden of disease and disability [18]. The likelihood of developing depressive disorders increases notably after puberty, especially among girls, who are 1.5 to 2 times more likely than boys to be diagnosed with depression. This gender difference persists throughout life [12, 19].

Worldwide, mental health disorders are among the top contributors to disability-adjusted life years (DALYs) in adolescents, imposing significant emotional, social, and economic strains. Research consistently reveals that conditions such as depression, anxiety, and conduct disorders are widespread among teenagers [20, 21]. However, the impact of these mental health issues is often overlooked compared to more visible health problems like infectious diseases and cardiovascular conditions. Although our study did not provide specific evidence, research in Malawi has shown that the overall prevalence of mental health disorders is 5.4% among children aged 6 to 12 years and 7.9% among adolescents aged 13 to 17 years [22].

During adolescence, the brain undergoes significant and dynamic development, contrasting the outdated view of it as a static organ [23]. However, the frequent occurrence of mental health issues during this period has not been thoroughly explored. With 40% of mood disorders having a genetic component, it’s crucial to consider societal factors, such as gender norms, and environmental influences, like hormonal changes during puberty, to fully understand the variations in mental health among adolescents [19]. On the other hand, mental health issues in adolescents are associated with poor academic performance, physical illness, substance abuse, and behavioral issues in later life [3]. The worldwide consequences of adolescent mental health distress underscore the necessity of pinpointing change mechanisms to guide efficacious therapies and the promotion of psychological well-being for this demographic [24]. The neglect of mental health issues, such as intellectual and developmental disorders, among children and adolescents living in low-resource environments is a public health concern that has far-reaching effects because it obstructs the attainment of fundamental developmental objectives [25].

Adolescents in low- and middle-income countries, particularly in Africa, often face neglected mental health needs despite these issues being a significant source of health-related disabilities in this age group, with enduring consequences. Current research highlights a varying prevalence of mental health distress among African adolescents, with several studies offering diverse findings. However, there has been no comprehensive systematic review or meta-analysis to consolidate this data into a unified estimate of the prevalence and risk factors associated with adolescent mental health distress in Africa. This systematic review and meta-analysis aim to fill this gap by providing a pooled estimate of the prevalence and identifying key risk factors contributing to mental health distress among African adolescents.

## Methods and materials

### Searching strategies

This systematic review and meta-analysis examined mental health distress among adolescents in Africa, following the PRISMA statement guidelines [26] (supplementary material). The study was registered in PROSPERO with the reference number CRD42023494665. It synthesized findings from original studies that explored the prevalence, incidence, and risk factors of mental health distress in African adolescents. To identify relevant published articles up to December 2023, comprehensive searches were conducted across multiple electronic bibliographic databases: PubMed, PsycINFO, Web of Science, Cochrane Library, Google Scholar, HINARI, and Science Direct. Search terms were aligned with Medical Subject Headings (MeSH) and combined using Boolean operators to ensure a thorough search. The search strategy incorporated key terms such as “mental health distress,” “emotional disturbance,” “distress,” “stress,” and “mental health problem,” paired with population terms like “teenagers” and “adolescents.” Additionally, terms related to geographical and epidemiological aspects such as “prevalence,” “risk factors,” and “psychological suffering in Africa” were included. Boolean operators (e.g., OR and AND) such as ((prevalence) OR (magnitude) OR ( burden) OR (epidemiology) OR (incidence)) AND ((determinants) OR (risk factors) OR (associated factors) OR (predictors)) AND ((mental health distress) OR (emotional disturbance) OR (psychological distress) OR (distress) OR (stress)) AND ((adolescents) OR (teenagers)) AND (Africa) were employed to refine the search and capture all relevant studies on the prevalence and risk factors of mental health distress among adolescents in Africa.

### Eligibility criteria

#### Inclusion criteria

This review covered research publications from nations in Africa. Original data indicating the frequency of mental health distress and risk factors in adolescents from observational studies (cross-sectional, case-series, and cohort studies) were included. Only articles published in the English language are included. Adolescents who had mental health distress were the study population. Published and grey pieces of literature available until December 2023 were incorporated.

#### Exclusion criteria

Excluded from consideration were incomplete studies, studies conducted outside of Africa, records without relevant results, editorial comments, letters to the editor, systematic reviews, and qualitative research.

#### Data extraction methods and quality assessment

All the articles retrieved from the searches were imported into EndNote version X7 software. Duplicate entries were identified and removed. Following this, three authors independently screened the articles to identify eligible studies based on the predefined inclusion criteria. The data extraction took place from 18/12/2023 to 18/01/2024. The extracted pieces of information included: the name of the author, years of publication, study population, study design, study setting, sample size, response rate, and prevalence and risk factors of mental health distress among adolescents in Africa. Data from all studies that fulfilled the inclusion criteria were extracted and tabulated. After an initial screening of the titles and abstracts, full the texts of eligible publications were reviewed. The arguments about the inclusion and interpretation of data were resolved by discussion between the reviewers. After the removal of duplications, from 1221 studies, eighteen relevant articles were selected for full-text analysis. The Newcastle-Ottawa Scale (NOS) criterion was used to evaluate the listed papers’ quality [27]. The agreement among three reviewers was assessed using both actual agreement and the unweighted Kappa statistic to gauge agreement beyond chance. Articles were evaluated based on a scoring system across three key categories for cross-sectional studies: selection (0–5 points), comparability (0–2 points), and outcome (0–3 points), resulting in a potential total score ranging from 0 to 9 [28].

The selection category encompassed criteria such as the adequacy of sample size, the representativeness of the sample, the rate of non-responses, and the use of validated measurement tools to gather exposure data. Comparability focused on determining whether subjects across different outcome groups were comparable based on study design and analysis, including the control of confounding factors. In the outcome category, reviewers examined whether outcome data were collected through independent blind assessment, from records, or via self-reporting. Additionally, they assessed whether the statistical methods used for data analysis were clearly described and appropriate for the study. Studies were categorized into two groups based on their total quality scores: those scoring 5 or less, and those scoring greater than 5. Reviewers’ assessments were categorized according to specified Kappa values: 0 for poor agreement, 0.01–0.20 for slight agreement, 0.21–0.40 for fair agreement, 0.41–0.60 for moderate agreement, 0.61–0.80 for substantial agreement, and 0.81–1.00 for almost perfect agreement. These evaluations provided a comprehensive framework for assessing the quality and consistency of the reviewers’ assessments across the evaluated articles [29].

### Outcome of interest

The outcome of this systematic review and meta-analysis was to estimate the pooled prevalence and risk factors of mental health distress among adolescents in Africa.

### Data analysis

We did a systematic review and meta-analysis of the reported prevalence and risk factors of mental health distress among adolescents in Africa. Estimated pooled prevalence and associated risk factors of mental health distress were reported based on the random effect model. In the included studies, heterogeneity was quantified using the I^2^ statistics [30]. The authors considered I^2^ values > 50% to represent significant heterogeneity. The values of heterogeneity in the included studies were > 95%. Due to this, we used the random effect model to get the estimated pooled prevalence of mental health distress in adolescents. Thus, the estimated pooled prevalence was reported in the form of percentages with a 95% CI. Furthermore, we did a subgroup analysis by grouping the measurement tools into three categories (K10, GSHS, and others). In addition, we did a subgroup analysis on the mean age of adolescents (older adolescents = 15–19 years; younger adolescents = 10–14 years). We evaluated publication bias using the funnel plot and Egger’s test to represent the bias graphically. For all analyses, we used STATA Version 11.

## Results

### Characteristics of studies

**Fig. 1:**
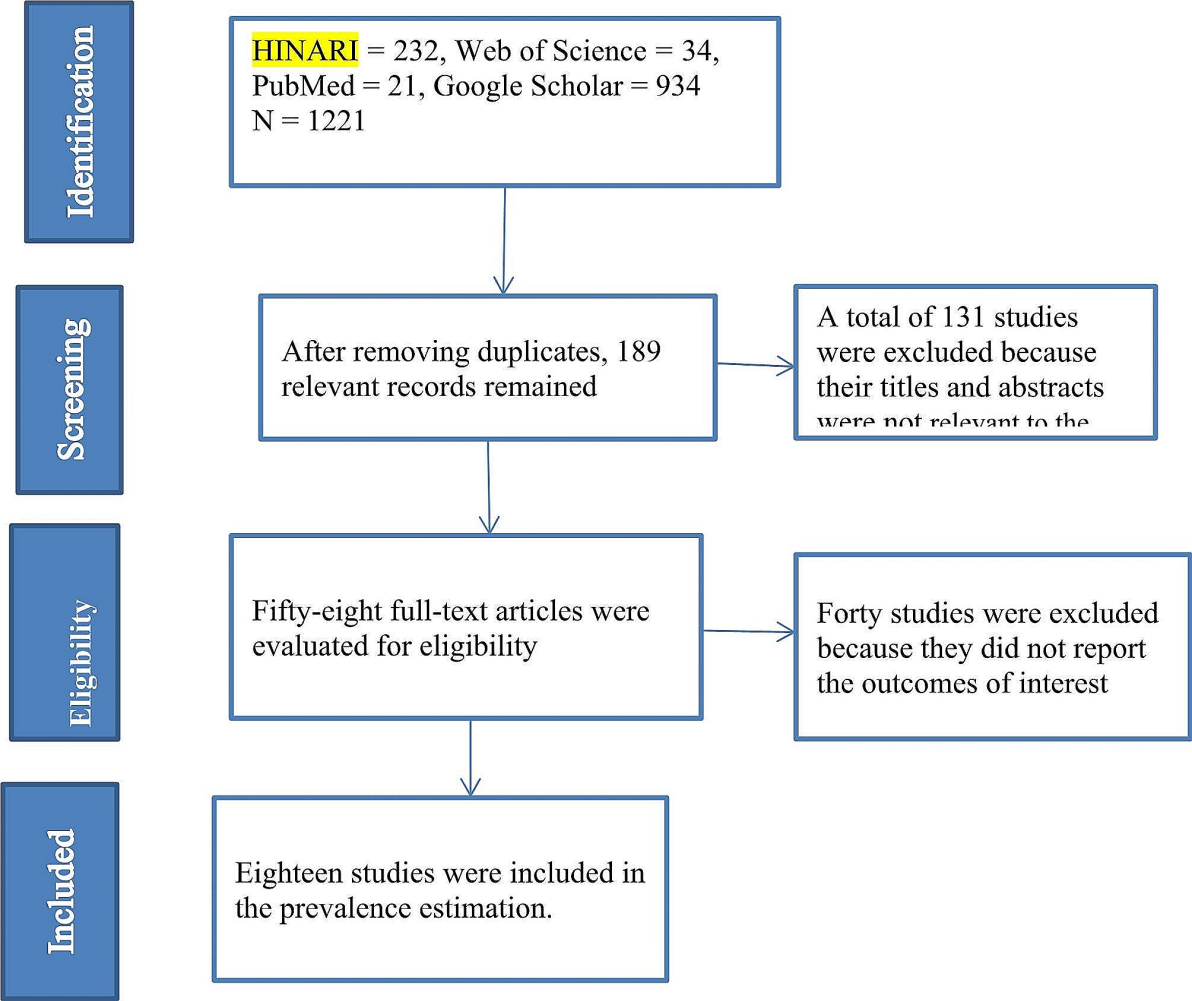
PRISMA flow chart showing the selection process of eligible studies for this review

We identified 1221 distinct article titles out of the 1298 items our search turned up. Of these, we evaluated 189 pertinent abstracts, determined the eligibility of 58 full-text publications, and included 18 papers in our final analysis. Data from 38,281 adolescents in total were included in the investigations (Fig. 1). In total, studies included in this review were conducted in ten African countries: Ethiopia [31–33], Tanzania [34], Uganda [35], South Africa [36], Nigeria [37–39], Liberia [40, 41], Benin [42, 43], Zamia [44], Mozambique [45], and Morocco [46]. The majority of the studies were conducted in Ethiopia. A greater number of papers (17 studies) were released in the 2020–2023 and three articles in the 2015–2019 timeframes. The mean age of the adolescents involved in the 10 studies was ≥ 15 years old; 6 researchers did not state the mean age, and 2 articles’ mean age was < 15 years. The most widely used research tool was the Global School-Based Student Health Survey (GSHS) (Table 1).

**Table 1:** Characteristics of included studies among adolescents with mental health distress, in Africa (*n* = 18)

Authors	Publication year	Countries	Mean age	Tool	Sample size	Prevalence (%)	Response rate (%)
Tarafa et al., 2021	2021	Ethiopia	14.9 years	K10	819	43.7	96.70
Mekonen et al., 2020	2020	Ethiopia	15 years old.	GHQ-12	405	55.1	95.90
Gebremariam et al., 2023	2023	Ethiopia	16.9 year	K10	377	44.56	96.20
Siziya and Mazaba, 2015	2015	Zambia	16 years	GSHS	2257	15.7	N/R
Anyanwu, 2023	2023	Uganda	16.9 years	K10	906	57	N/R
Amu et al., 2020	2020	Mozambique	N/A	GSHS	1918	21.2	80
Pengpid and Peltzer, 2021	2021	Liberia	18 years, median age	GSHS	2635	24.5	N/R
Mwakanyamale et al., 2022	2022	Tanzania	16.44 years	K10	3193	25.84	N/R
Pengpid and Peltzer, 2020a	2020a	Morocco	15 years, median	GSHS	6745	23.3	N/R
Akanni and Otakpor, 2016	2016	Benin	N/R	GHQ-28	376	30.9	91.30
Lebimoyo and Olibamoyo, 2023	2023	Nigeria	N/R	RSES	107	20	100
Akadri et al., 2022	2022	Nigeria	N/R	GHQ-12	212	34.9	84.10
Pengpid and Peltzer, 2021	2021	Liberia	18 years, median age	GSHS	2744	24.5	N/R
Pengpid and Peltzer, 2022	2022	South Africa	17 years, median age	GSHS	2240	16	N/R
Olga et al., 2019	2019	Benin	16.54 years	census	3953	10.8	78.00
Pengpid and Peltzer, 2020	2020	Tanzania	N/R	GSHS	3765	20.6	87
Pengpid and Peltzer, 2020	2020	Tanzania	N/R	GSHS	3765	10.3	87
Ozoalor et al., 2022	2022	Nigeria	N/R	YPSC	4364	17.6	N/R

N/R*= not reported

GHQ-12^*^= General health questionnaire

GHQ-28^*^= General health questionnaire

GSHS^*^= Global school-based student health survey

K-10^*^= Kessler Mental Health Distress Scale

RSES^*^ =Rosenberg Self-Esteem Scale

YPSC*= Youth version of the Pediatric Symptom Checklist

### Quality of included studies

In our quality evaluation, we found that all the included studies had a reputable methodological quality (NOS) score ranging from 7 to 9 from a total 9-point) (Table 2).

**Table 2:** Quality assessments in the included studies in this meta-analysis and systematic review

Author	Selection	Comparability	Outcome	NOS total score (0 to 9)	Kappa value	95% CI for Kappa
Tarafa et al. (2021)	4	2	3	9	1	0.99-1.00
Mekonen et al. (2020)	4	2	3	9	1	0.99-1.00
Gebremariam et al. (2023)	4	2	3	9	1	0.99-1.00
Siziya and Mazaba (2015)	3	2	3	8	0.88	0.72–0.98
Anyanwu (2023)	4	2	2	8	0.88	0.70–0.92
Amu et al. (2020)	4	2	3	9	1	0.99-1.00
Pengpid and Peltzer (2021)	3	2	3	8	0.88	0.72–0.98
Mwakanyamale et al. (2022)	3	2	3	8	0.88	0.72–0.98
Pengpid and Peltzer (2020a)	4	2	2	8	0.88	0.70–0.92
Akanni and Otakpor (2016)	3	2	3	8	0.88	0.72–0.98
Lebimoyo and Olibamoyo (2023)	3	2	3	8	0.88	0.72–0.98
Akadri et al. (2022)	3	2	3	8	0.88	0.72–0.98
Pengpid and Peltzer (2021)	4	2	2	8	0.88	0.70–0.92
Pengpid and Peltzer (2022)	3	2	3	8	0.88	0.72–0.98
Olga et al. (2019)	4	2	3	9	1	0.99-1.00
Pengpid and Peltzer (2020)	3	2	3	8	0.88	0.72–0.98
Pengpid and Peltzer (2020)	3	2	3	8	0.88	0.72–0.98
Ozoalor et al. (2022)	3	2	2	7	0.77	0.60–0.90

### The pooled estimated point prevalence of mental health distress among adolescents dwelling in Africa

**Fig. 2:**
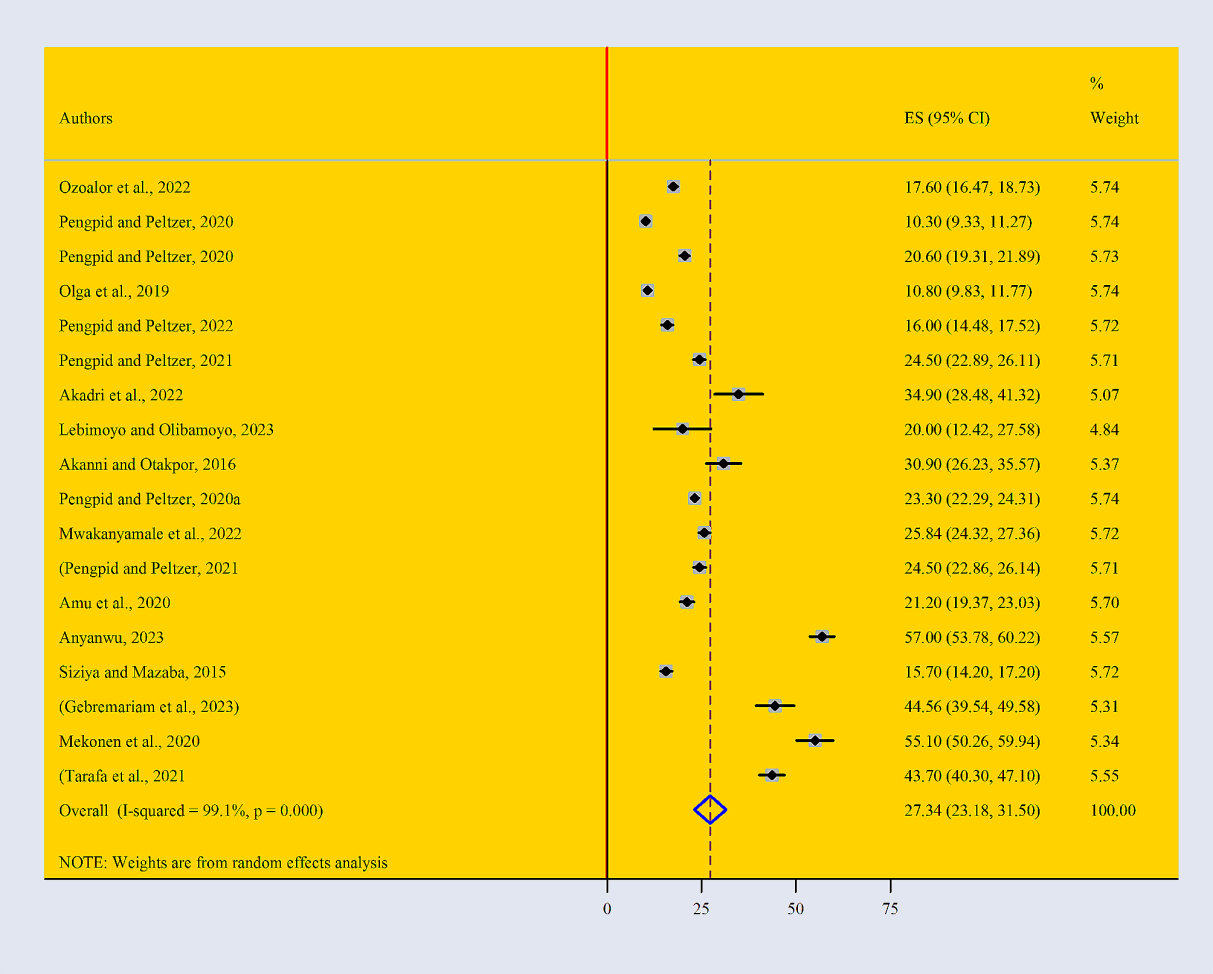
Forest Plot describing the pooled prevalence of mental health distress among adolescents in Africa with a 95% CI

Based on the random effect model, the pooled burden of mental health distress among adolescents in Africa was 27.34% (95% CI: 23.18–31.50) (Fig. 2). According to the finding of current study, the higher prevalence of mental health distress is found among older adolescents (29.44% with a 95% CI: 23.26–35.66) compared with younger adolescents (42.74% with a 95% CI: 26.60–59.87). We found significant heterogeneity between studies (I2 = 99.1%, *P* = 0.000). Because of the high heterogeneity subgroup analysis was conducted.

### Publication bias

**Fig. 3:**
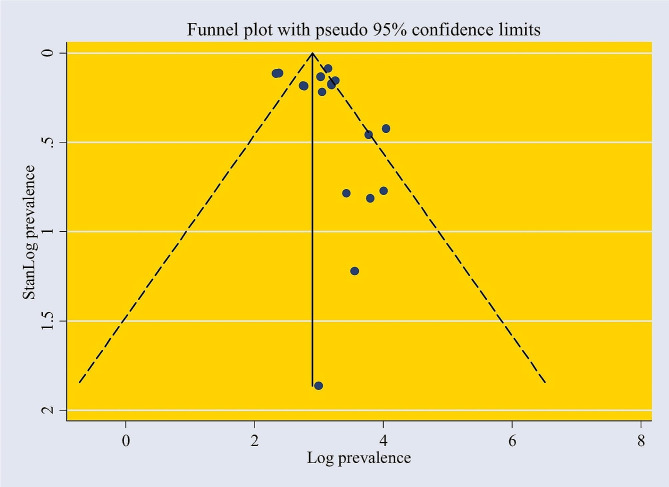
Funnel plot with a pseudo95% confidence interval that investigated the heterogeneity of the pooled prevalence of mental health distress among adolescents in Africa

**Fig. 4:**
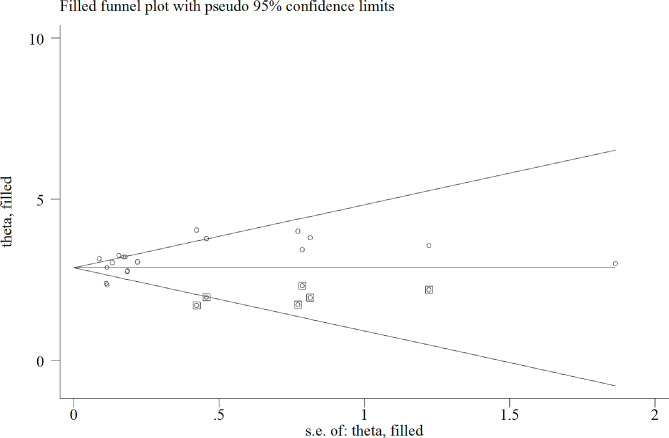
Trim and fill analysis of mental health distress among adolescents in Africa

Based on the funnel plot, the test of publication bias revealed asymmetric distributions (Fig. 3). In addition, Egger’s test shows a significant bias coefficient (*p* = 0.003). This suggests the presence of publication bias. So, a trim and fill analysis was conducted to manage the publication bias (Fig. 4). The sensitivity analysis shows the estimated pooled values varied between 25.50 (21.80-29.19) and 28.37 (24.16–32.57) after the deletion of a single study (Table 3).

**Table 3:** Sensitivity analysis of the included studies to the pooled prevalence of mental health distress among adolescents

Omitted articles	Prevalence of mental health distress among patients living in East African countries (95% CI)
Tarafa et al. (2021)	26.35 (22.26–30.44)
Mekonen et al. (2020)	25.75 (21.69–29.81)
Gebremariam et al. (2023)	26.36 (22.18–30.54)
Siziya and Mazaba (2015)	28.06 (23.64–32.48)
Anyanwu (2023)	25.50 (21.80-29.19)
Amu et al. (2020)	27.72 (23.34–32.10)
Pengpid and Peltzer (2021)	27.52 (23.15–31.89)
Mwakanyamale et al. (2022)	27.44 (23.09–31.78)
Pengpid and Peltzer (2020a)	27.61 (23.08–32.14)
Akanni and Otakpor (2016)	27.13 (22.87–31.40)
Lebimoyo and Olibamoyo (2023)	27.71 (23.44–31.98)
Akadri et al. (2022)	26.93 (22.68–31.19)
Pengpid and Peltzer (2021)	27.52 (23.15–31.89)
Pengpid and Peltzer (2022)	28.04 (23.62–32.46)
Olga et al. (2019)	28.34 (24.09–32.60)
Pengpid and Peltzer (2020)	27.37 (23.28–32.57)
Pengpid and Peltzer (2020)	28.37 (24.16–32.57)
Ozoalor et al. (2022)	27.96 (23.41–32.51)

### Subgroup analysis

**Fig. 5:**
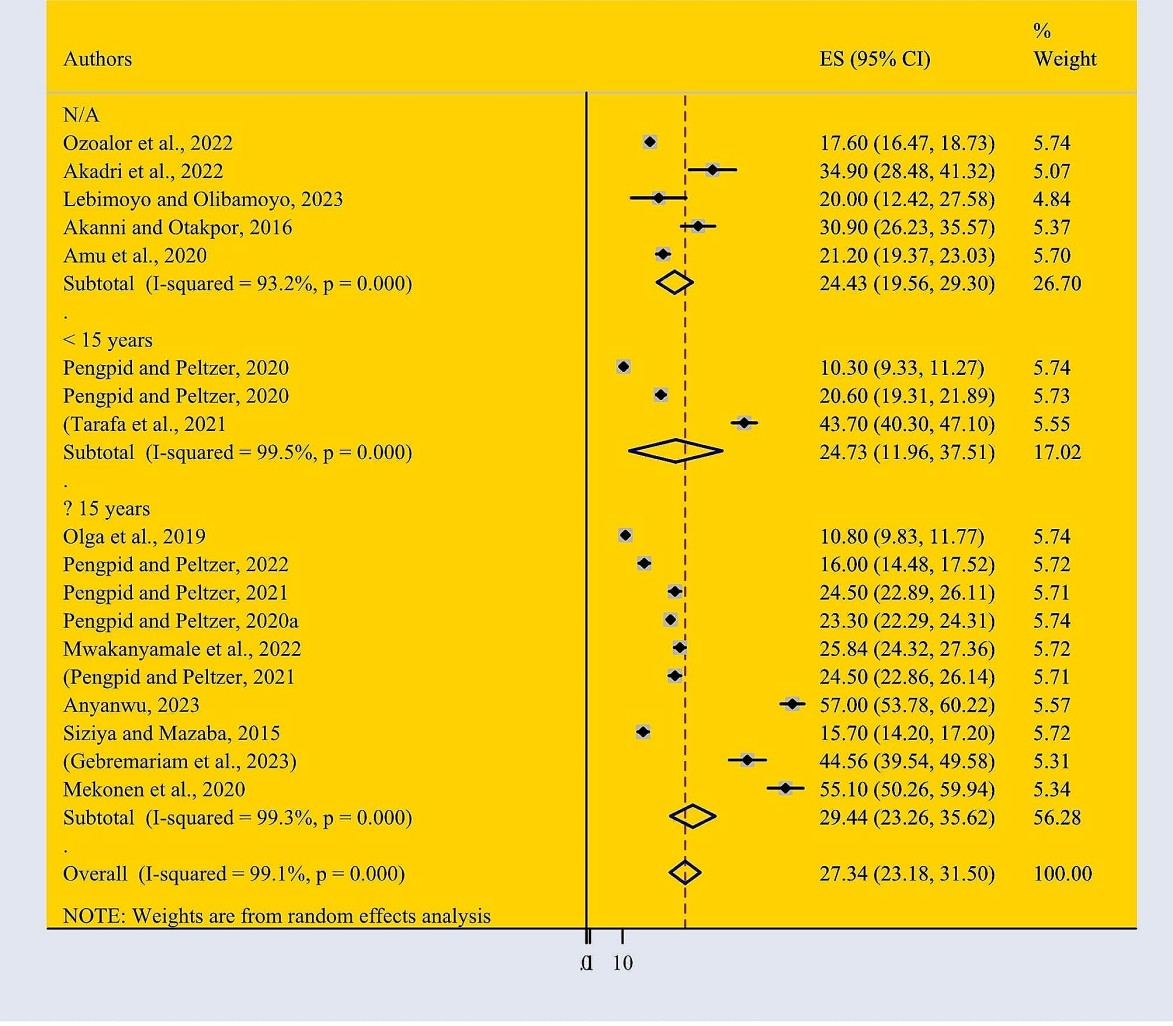
Forest Plot describing the sub-group analysis based on age in Africa with 95% CI

**Fig. 6:**
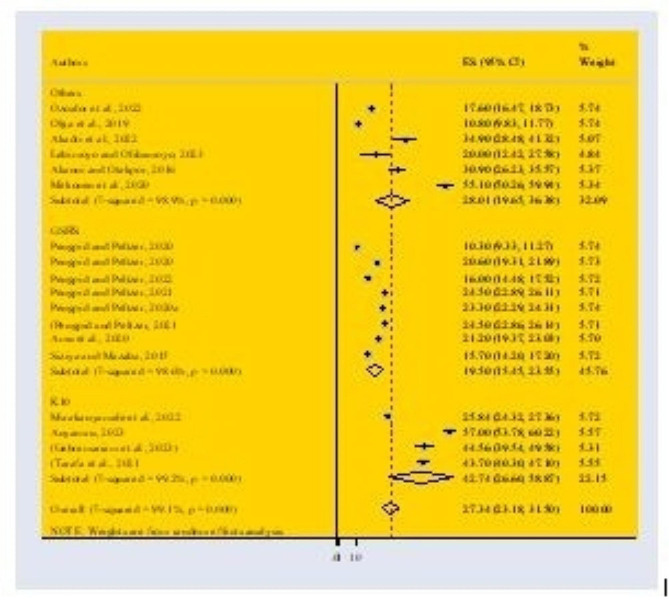
Forest Plot describing the sub-group analysis based on measurement tools in Africa with 95% CI.  Others*= GHQ-28, GHQ-12, RSES, YPSC, and Census

Subgroup analysis demonstrates the estimated prevalence of mental health distress among older adolescents (≥ 15 years) (pooled prevalence = 29.44%; 95% CI: 23.26–35.66) and younger adolescents (< 15 years) (pooled prevalence = 24.73%; 95% CI: 11.96–37.51) respectively (Fig. 5). In addition, another Subgroup analysis was conducted based on measurement tool which demonstrates the estimated prevalence of mental health distress using K10 as a measurement tool (pooled prevalence = 42.74%; 95% CI: 26.60-59.87) and GSHS (pooled prevalence = 19.50%; 95% CI: 15.45–23.55) (Fig. 6).

### Associated factors

**Fig. 7:**
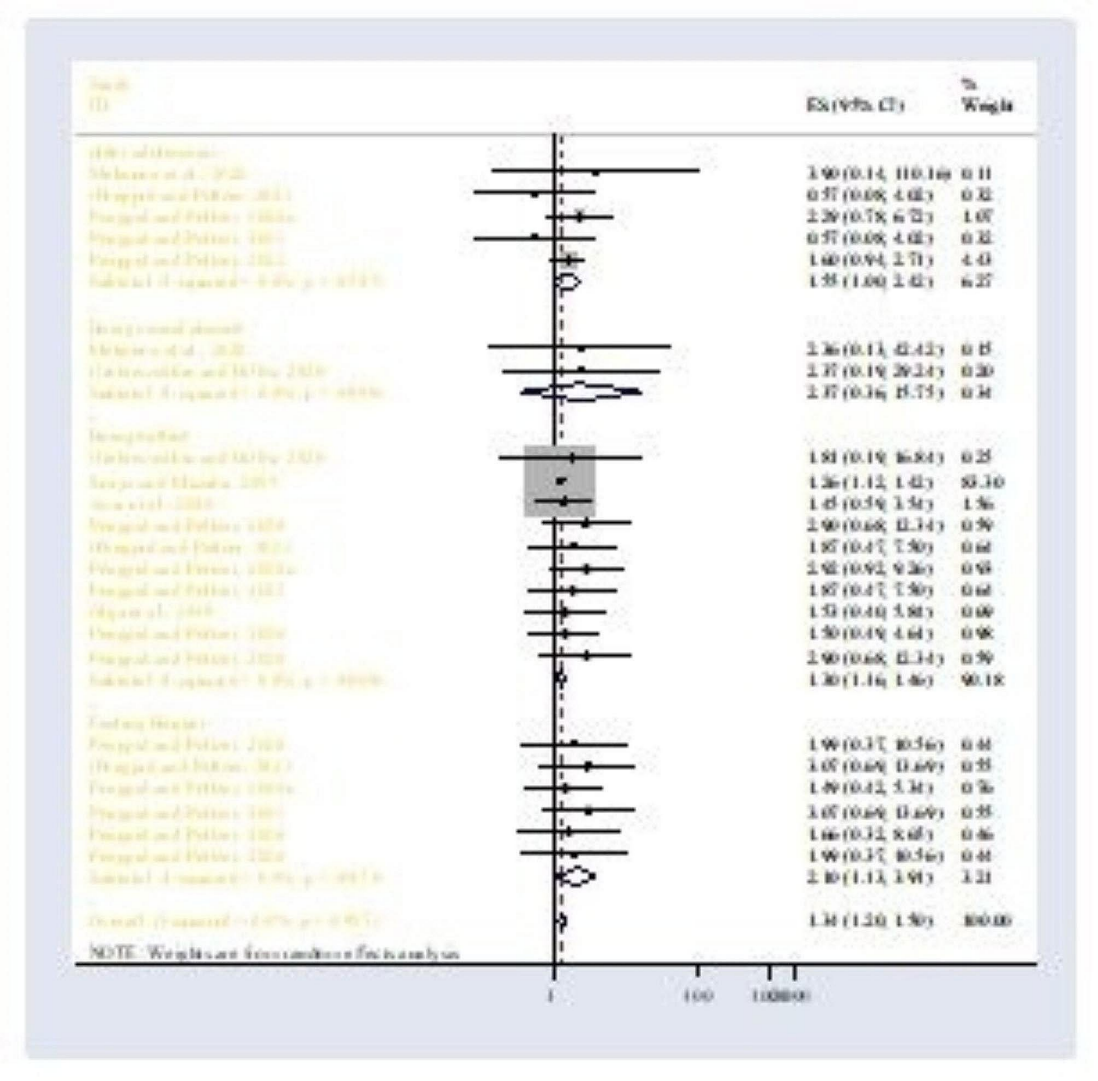
Factors associated with mental health distress among adolescents in Africa

Of eighteen included studies only nine studies were eligible to analyze risk factors for mental health distress among adolescents in Africa. Being sexually abused, age greater or equal to 15 years, bullying victimization, and experiencing feelings of hunger were the included variables. Thus, based on the random effect model, the following factors were associated as risk factors for mental health distress among adolescents. The odds of bullying victimization (POR = 1.30; 95%: 1.16, 1.46), and the odds of experiencing hunger (POR = 2.10; 95% CI: 1.13, 3.91) were the risk factors for mental health distress (Fig. 7).

## Discussion

This meta-analysis is the first attempt to ascertain the pooled point prevalence and risk factors of mental health distress among adolescents in Africa. Because mental health problems in the world among adolescents have been steadily increasing over the last decades [47]. Despite the high prevalence and related risk factors causing mental health-related disabilities among this age group the pooled prevalence and leading risk factors of mental health distress and the mental health needs among adolescents are neglected in low- and middle-income countries in particular. In the current meta-analysis, we tried to review the pooled prevalence of mental health distress and its determinant factors to fill the mental health evidence gap in this age group. In this review 22 published studies were pooled together to assess the prevalence and risk factors of mental health distress among adolescents dwelling in Africa. According to our research, the prevalence of mental health distress among adolescents in Africa was 27.34% (95% CI: 23.18–31.50). bullying victimization and experiencing feelings of hunger were the major risk factors for mental health distress.

According to this meta-analysis, the prevalence of mental health distress was comparable to previous studies done in China reporting a 27% prevalence of mental health distress in adolescents [48]. We did not get similar compiled work, which needs to be compared with these findings. However, the result of this finding is lower compared to previous studies which reported a prevalence of mental health distress of 32%, 35%, 50.0%, and 61.97% [49–52]. The possible justification for this discrepancy might be the existing previous studies, were single studies but in our study, we assessed the pooled prevalence of mental health distress. This may lower the magnitude of mental health distress among adolescents. In the controversial, this finding is significantly higher compared with previous studies done among adolescents in Vietnam, accounting for 5.4%, in India at 5.42%, and in multicounty studies at 13% [53–55] respectively. This discrepancy might be due to mental health service access differences, family awareness about the adolescent stage, and socioeconomic differences.

We did a subgroup analysis with adolescents’ age categories (greater or equal to fifteen years and less than fifteen years), and those whose ages were ≥ 15 years showed higher mental health distress than those whose ages were < 15 years. These results are in line with other studies showing that older adolescents experienced significant emotional and behavioral problems [56–59] than younger adolescents. This disparity may be because of the biological, psychological as well as social relationship differences between these two stages of adolescents. As age increases older adolescents might be exposed to different stressors such as academic pressures, peer relationships, future uncertainties (college, career), and familial expectations. These stressors can exacerbate emotional disturbances. Older adolescents are in the process of forming their identity, which involves grappling with existential questions, self-discovery, and defining their values and beliefs. This process can lead to emotional turmoil and confusion, Puberty brings about significant hormonal changes, which can impact mood regulation and emotional stability. Older adolescents may face peer pressure, social comparison, and conflicts within friendships and romantic relationships, which can contribute to emotional disturbances. As adolescents grow older, they seek greater autonomy from their parents and authority figures. Balancing newfound independence with responsibility can be challenging and may lead to feelings of anxiety or stress. Older adolescents may face stigma or reluctance to seek help for mental health issues due to societal attitudes or perceptions of weakness, which can exacerbate emotional disturbances by delaying or preventing access to appropriate support and treatment. Overall, the combination of biological, psychological, social, and environmental factors contributes to the higher prevalence of mental health disturbance among older adolescents compared to younger ones.

Regarding risk factors associated with mental health distress among adolescents. Adolescents who had a history of bullying victimization were 1.3 times more vulnerable to mental health distress than their counterparts. This finding is supported by similar studies that reveal that the behavior of bullying represents a risk factor for mental health distress in adolescents [60–63]. This may be because bullying can cause feelings of rejection, isolation, low self-esteem, and exclusion and some individuals may develop depression, anxiety, and low confidence. Being repeatedly subjected to aggressive behavior can lead to a range of psychological issues such as anxiety, depression, low self-esteem, and feelings of helplessness. Adolescents may internalize the negative messages conveyed through bullying, leading to a distorted self-perception and emotional turmoil. Victims of bullying often experience social isolation as they may withdraw from social interactions out of fear or humiliation. This isolation can exacerbate feelings of loneliness and further contribute to mental distress. Victims of bullying may develop distorted thinking patterns, such as negative self-talk and a pervasive sense of hopelessness. These cognitive distortions can perpetuate feelings of distress and contribute to the development of mental health disorders. Adolescents who are bullied may feel a lack of support from peers, teachers, or family members, which can exacerbate their sense of isolation and distress. A lack of intervention or support from adults in authority can also contribute to a feeling of powerlessness and further amplify the impact of bullying on mental health. Therefore, effective prevention strategies are crucial for addressing bullying and mitigating its effect on adolescents’ mental health and well-being.

In addition, adolescents with a history of experiencing hunger or feeling hunger twice increase the risk of mental health distress. This finding is supported by previous studies [63–65]. This may be a feeling of hunger that can cause worrying about not having enough food; worrying about their parents’ well-being, anger and irritation about not having enough food, humiliation about their family’s food status, strain on the family’s relationships due to food insecurity, and sadness about not having enough food. Adolescents often face significant stressors, such as academic pressure, social challenges, and family dynamics. Stress and anxiety can affect appetite, leading to fluctuations in hunger levels. In some cases, individuals may experience a decrease in appetite due to stress, while others may seek comfort in food, leading to increased hunger. Depression can manifest in different ways, including changes in appetite. Some adolescents may experience increased hunger as a coping mechanism to alleviate emotional distress. On the other hand, others may lose their appetite due to feelings of sadness or hopelessness. Mental health disorders can impact the regulation of hormones and neurotransmitters involved in appetite control. Imbalances in serotonin, dopamine, and other neurotransmitters can influence hunger cues and eating behaviors. Socioeconomic status, cultural influences, and access to nutritious food can also play a role in the relationship between mental health and hunger among adolescents. Food insecurity, for example, can exacerbate stress and contribute to disordered eating patterns.

### Limitations and strengths

This study has several strengths: To reduce the potential for assessor bias, we first followed a predetermined protocol for the search strategy and data abstraction and carried out a quality assessment by two separate investigators; second, we performed subgroup and sensitivity analyses based on the instrument used and the age of the research participant. However, the limitation of this study is its narrow scope, which included studies involving only adolescents and African countries. Moreover, all of the studies in this systematic review and meta-analysis had cross-sectional study designs. Another limitation of the present study is the language limitation.

## Conclusion

Mental health distress is a highly prevalent mental health problem among adolescents in Africa. Bullying victimization and feelings of hunger were the risk factors for mental health distress in adolescents. More funding must be set aside for the African adolescent mental health care system’s capacity-building. Itis essential to take action to lessen the burden of mental health issues in the coming generations and to enable adolescents to reach their full potential. This study can raise awareness about the mental health distress of adolescents among their teachers and parents and guide them to take the necessary interventions.

### Electronic supplementary material

Below is the link to the electronic supplementary material.

Supplementary Material 1

## Data Availability

No datasets were generated or analysed during the current study.
